# Prostate squamous cell carcinoma developing 11 years after external radiotherapy for prostate adenocarcinoma

**DOI:** 10.1002/iju5.12161

**Published:** 2020-04-20

**Authors:** Toru Matsugasumi, Hiroyuki Nakanishi, Tomohiro Yokota, Takumi Shiraishi, Osamu Ukimura

**Affiliations:** ^1^ Department of Urology Kyoto Chubu Medical Center Nantan City Kyoto Japan; ^2^ Department of Urology Kyoto Prefectural University of Medicine Kyoto Japan

**Keywords:** prostate‐specific antigen, radiotherapy, secondary prostate cancer, squamous cell carcinoma

## Abstract

**Introduction:**

Secondary bladder, colon, and rectal cancers are relatively common after prostate radiotherapy. However, secondary squamous cell carcinoma of the prostate is rare.

**Case presentation:**

An 85‐year‐old man presented with dysuria and low‐serum prostate‐specific antigen levels. His medical history included localized prostate adenocarcinoma (Gleason score of 4 + 5) treated with combined three‐dimensional conformal radiotherapy and androgen deprivation therapy, 11 years ago. Urethroscopy and magnetic resonance imaging showed a bulging mass around the prostatic urethra. Transurethral resection of the prostate performed for histopathological diagnosis revealed squamous cell carcinoma.

**Conclusion:**

Hereby, a rare case of secondary squamous cell carcinoma of the prostate after radiotherapy for adenocarcinoma was reported, which was found after 11 years of radiotherapy with symptom of dysuria including urinary hesitancy, difficulty, pain during urination, and low‐serum prostate‐specific antigen levels.

**Abbreviations & Acronyms**
ADT: androgen deprivation therapy
PSA: prostate‐specific antigen
SCC: squamous cell carcinoma
TURP: transurethral resection of the prostate

**Keynote message**

We reported a case of secondary SCC of the prostate after radiotherapy for adenocarcinoma was reported, which was found after 11 years of radiotherapy with symptom of dysuria including urinary hesitancy, difficulty, pain during urination, and low‐serum PSA levels. SCC of the prostate is rare and poor prognosis. SCC of the prostate on the effect of radiotherapy might be considered in a patient with dysuria and low‐serum PSA levels.

## Introduction

Radiation therapy is a firmly established treatment for prostate cancer, in view of developing new technologies and safety. However, there are several issues with prostate radiation therapy including rectal disorders, dysuria, and other secondary neoplasms. SCC of the prostate is a rare neoplasm that accounts for 0.5%–1.0% of the total number of prostate cancer cases.^1^ Herein, we report a case of prostate SCC occurring 11 years after radiation therapy and ADT for prostate adenocarcinoma. The patient provided written informed consent and was guaranteed confidentiality.

## Case presentation

An 85‐year‐old man visited our hospital with dysuria including urinary hesitancy, difficulty and pain during urination, and hematuria. He had no family history of cancer, including prostate cancer, and had undergone a prostate biopsy 13 years earlier owing to an elevated PSA level of 7.18 ng/mL. Pathological evaluation revealed an adenocarcinoma with a Gleason score of 4 + 5 and a clinical stage of T3aN0M0. At that time, he elected to undergo 3D conformal radiation therapy up to a dose of 70 Gy combined with ADT for a total of 2.5 years. Thereafter, his serum PSA level dropped to 0.20–0.25 ng/mL for approximately 7 years.

**Fig. 1:**
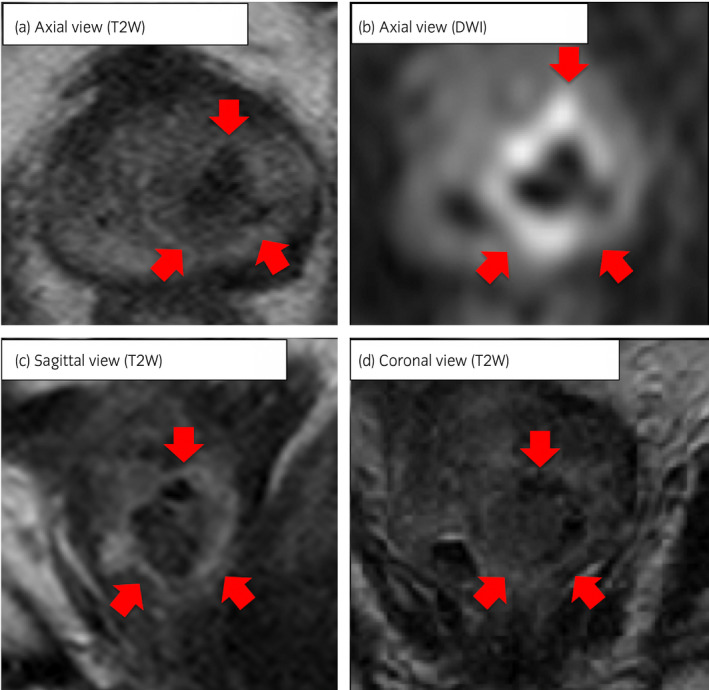
Pelvic magnetic resonance imaging showing an unclear boundary between the prostatic urethra and the transition zone, and the lesion showing signal intensity decreasing from the middle to the apex on T2‐weighted images (a, c, d). The same lesion showing an unclear boundary, and high signal intensity outside the lesion on the diffusion‐weighted image (b). Arrows showing an unclear boundary contour of the prostate SCC.

**Fig. 2:**
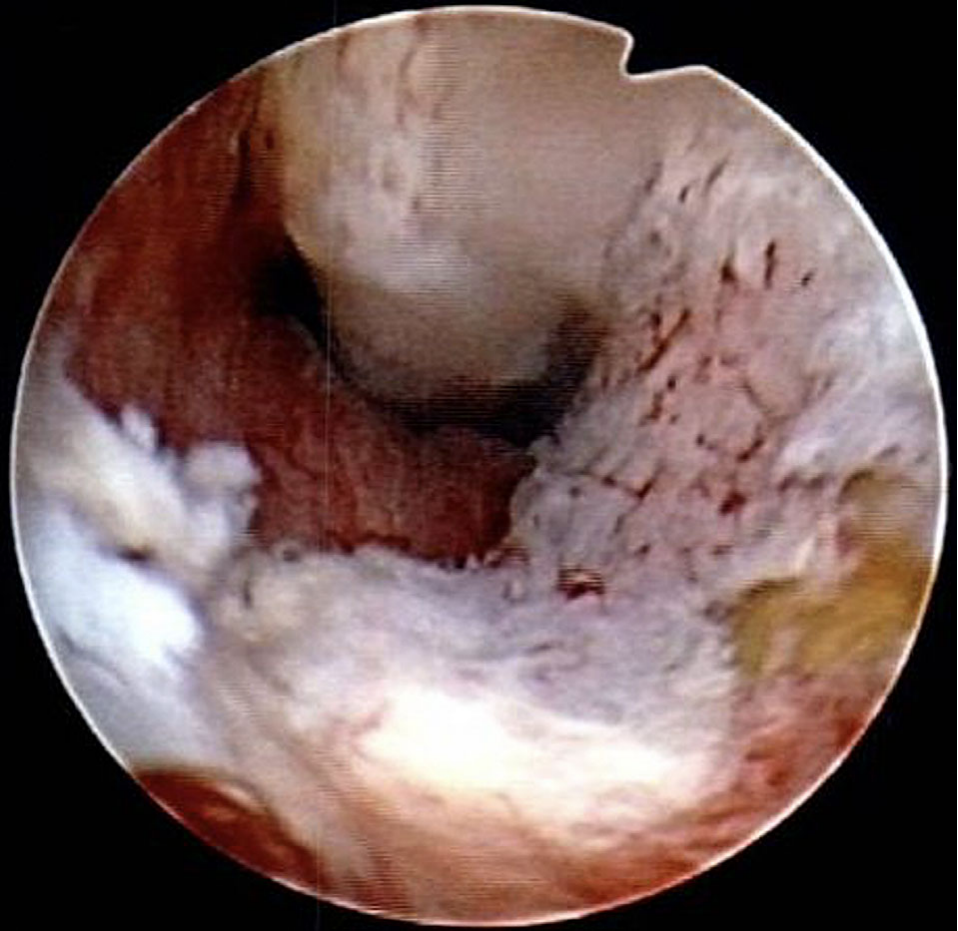
Urethrocystoscopy showing bulging irregular mucosa with hemorrhage in the prostatic urethra.

At the present visit, digital rectal examination revealed a swollen and enlarged prostate with a smooth surface; the PSA level was 0.41 ng/mL. Whole body computed tomography revealed no enlarged lymph nodes or visceral metastases; prostate magnetic resonance imaging showed an unclear boundary between the prostatic urethra and the transition zone border, and decreasing signal intensity from the middle to the apex (Fig. 1). Magnetic resonance imaging findings did not clearly indicate malignant disease. Urethrocystoscopy, performed to assess the prostatic urethra for hyperplasia or stenosis, revealed bulging irregular mucosa in the prostatic urethra (Fig. 2); urine cytology and culture were negative. The location of the lesion almost corresponded to that of the diagnostic positive biopsy.

**Fig. 3:**
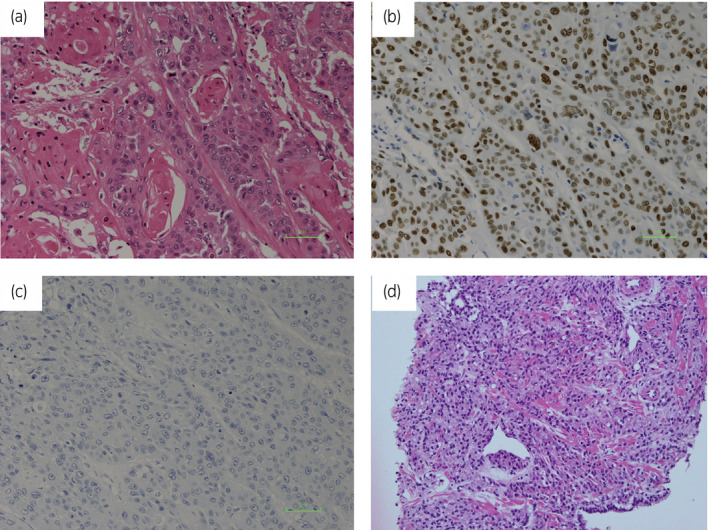
(a) Microscopic section of the prostate revealing a nest of malignant squamous cells with cytoplasmic keratinization on hematoxylin‐eosin staining (scale bar, 100 μm). (b) Microscopic section of the prostate revealing p40‐positive cells on immunohistochemical staining, indicative of SCC (scale bar, 100 μm). (c) Microscopic section of the prostate revealing PSA‐negative cells on immunohistochemical staining (scale bar, 100 μm). (d) Microscopic section of the prostate revealing adenocarcinoma with Gleason score 4 + 5 on the diagnostic biopsy on hematoxylin‐eosin staining.

TURP was subsequently performed for diagnosis as the PSA level was not elevated, and the differential diagnoses included radiation‐induced inflammatory responses or hyperplasia rather than malignancy on magnetic resonance imaging findings. Histopathological examination revealed SCC based on proliferation of atypical squamous epithelial cells with keratinization (Fig. 3a). Microscopic sections of the prostate revealed p40‐positive cells on immunohistochemical staining, indicative of SCC (Fig. 3b); however, the PSA recurrence was negative (Fig. 3c). The AJCC‐UICC stage of the SCC was stage II (T2cN0M0) and serum SCC level after TURP was 0.6 ng/mL (normal range: <1.5 ng/mL). PSA and SCC values were not increased at 0.16 and 0.7 ng/mL, respectively. Radiographic progression was also not seen for 3 months. He subsequently underwent total pelvic exenteration for severe prostatic pain and recurrent urinary retention owing to secondary cancer of the prostate. The pathological findings revealed that the SCC had invaded the subserosal layer of the rectum.

## Discussion

Radiation therapy is a radical treatment for localized prostate adenocarcinoma, and has been increasingly recommended as an alternative to surgery owing to its low invasiveness and excellent cure rates. However, an increased risk of subsequent secondary cancer has been reported.

Murray *et al*.^2^ described cancers developing in an irradiation field more than 5 years after initiating radiotherapy, with a tissue type different from that of the primary cancer, as secondary. They may be caused by genetic changes after radiation therapy. Our case fits the definition of secondary cancer after radiotherapy. Secondary cancer of the prostate was diagnosed as it was unclear whether the cancer was primary SCC or secondary, owing to recurrence. Secondary bladder, colon, and rectal cancers have been reported to be relatively common after prostate radiation therapy. However, secondary cancer of the prostate itself is rare.

Miller *et al*.^3^ reported a case of primary SCC of the prostate after radiation seed implantation for treating prostate adenocarcinoma. In that case, SCC was confirmed 8 years after seed implantation with elevated PSA levels (20 ng/mL). Agrawal *et al*.^4^ also reported another similar case, which was confirmed 4 years after brachytherapy, owing to retention. Many secondary cancers have been reported after radiotherapy; however, secondary SCC after radiotherapy is rare.

The treatment of primary and secondary prostate SCC does not differ, and their prognoses are poor.^5^, ^6^ The average survival with secondary cancer of the prostate remains unknown; however, the average survival of primary SCC of the prostate is 16–24 months;^2^, ^7^ the 1‐, 3‐, and 5‐year cancer‐specific survival rates are 55.2%, 37.8%, and 30.3%, respectively, and the 1‐year survival rate without treatment is 38.7%.^7^ SCC is believed to originate from squamous metaplasia of the acinar and ductal tissues; this occurs frequently in cases of chronic inflammation or infarction. Malignant tumors such as prostate adenocarcinoma that occur after endocrine therapy or radiotherapy show a wide range of differentiation into squamous epithelium. It has also been hypothesized that heterogeneous tumors originate from cancer stem cells, possessing the properties of self‐renewal, pluripotency, and tumorgenicity.

Arva and Das^6^ reported prostate SCC in 35 of 66 cases after either endocrine therapy or radiotherapy; however, whether these tumors were primary or secondary cancers was unclear. The diagnosis modalities were various, performed by prostate biopsy, TURP, prostatectomy, and cystoprostatectomy. SCC of the breast is rare, accounting for approximately 0.1% of all breast cancers. Only two cases of secondary breast cancer have been reported after radiotherapy,^8^, ^9^ occurring after 8 and 15 years, respectively. Our case occurred 11 years after irradiation; this duration was longer than that described by Arva and Das.^6^ Murray *et al.* ^2^ reported that the risk of radiation‐induced secondary cancer is likely to be small, ranging between 1 in 220 and 1 in 290 over all follow‐up durations; however, this may be increased to 1 in 70 for patients followed up for more than 10 years. These findings suggest that longer follow‐up is required for men who have undergone prostatic radiation therapy, as established evidence or follow‐up protocols are currently not available for adenocarcinoma. Hence, caution is required when recommending radiation therapy for relatively young men diagnosed with prostate cancer.

## Conclusion

Hereby, we describe a rare case of secondary SCC of the prostate after radiotherapy for adenocarcinoma, which was found after 11 years of radiotherapy with symptom of dysuria including urinary hesitancy, difficulty, pain during urination, and low PSA level.

## Conflict of interest

The authors declare no conflict of interest.
